# Influence of Compound Modification of Oil Sands De-Oiled Asphalt and Polyphosphoric Acid on High- and Low-Temperature Performance of Styrene-Butadiene-Styrene-Modified Asphalt

**DOI:** 10.3390/ma14040797

**Published:** 2021-02-08

**Authors:** Xiaoguang Pei, Weiyu Fan

**Affiliations:** State Key Laboratory of Heavy Oil Processing, China University of Petroleum, Qingdao 266580, China; b15030089@s.upc.edu.cn

**Keywords:** composite modified asphalt, rheological properties, storage stability, microstructure, oil sands de-oiled asphalt, polyphosphoric acid

## Abstract

Oil sands de-oiled asphalt (OSDOA) has become a bottleneck for refineries due to its enormous production and huge landfill costs. Applying OSDOA as a modifier is an effective way to reduce environmental pollution and disposal cost. In this study, the influences of OSDOA and polyphosphoric acid (PPA) compound modification on styrene-butadiene-styrene (SBS)-modified binder were investigated. The high-temperature rutting resistance, low-temperature anti-crack performance and fatigue resistance were obtained by dynamic shear rheometer (DSR) and bending beam rheometer (BBR) test. Storage stability and microstructure were also investigated by storage test and Fourier-transform infrared (FTIR) spectroscopy. The results demonstrated that the compound modification of OSDOA/PPA dramatically enhanced the deformation resistance of SBS-modified binder and reduced its low-temperature cracking resistance. The anti-fatigue performance was also decreased. Moreover, the combined effect of OSDOA and PPA could produce composite modified asphalt with excellent storage stability, which was verified by desirable fluorescence images. Furthermore, both physical and chemical interactions coexisted during the OSDOA/PPA compound modification process. Consequently, the optimal doses of OSDOA and PPA were determined to be 10 wt% and 1.0 wt%, considering of the balance between high- and low-temperature characteristics and storage stability of composite modified asphalt.

## 1. Introduction

Asphalt is a viscoelastic material with a long history of application in pavement and is difficult to replace due to its abundant sources and low price [1,2]. Currently, asphalt pavement has been developed with advantages of decreasing the urban heat island effect, reducing tires noise, reducing the surface run-off and the spray effect, leading to a safer driving [3,4,5]. Unfortunately, pavements containing neat asphalt are becoming overwhelmed with increasing traffic volume, overloaded vehicles, and extreme weather conditions [6,7]. The frequent occurrence of pavement distress leads to a shortening of pavement in-service life and a growth in maintenance costs [8]. To alleviate pavement deterioration, high-performance types of polymer-modified binder have been applied in a wide range of situations. Among them, styrene-butadiene-styrene (SBS)-modified asphalt has been broadly used in road engineering because of its excellent pavement performance [9,10]. However, the performance of modified asphalt depends to some extent on its asphalt source and the concentration of SBS [11,12]. Lower SBS concentrations may lead to poor performance, but higher concentrations contribute to the higher costs and lower workability. Therefore, to decrease the costs and improve the property of modified binder it is essential to partially replace SBS with other modifiers [13].

De-oiled asphalt (DOA), a by-product of solvent deasphalting (SDA), has a high softening point and low penetration [14]. In general, it is mainly applied in the production of paving asphalt and construction asphalt [15]. Literature reveals that paving asphalt can be produced by blending DOA and fluid catalytic cracking slurry [16,17]. DOA increases the rutting resistance, water stability, and thermal storage stability of asphalt mixtures [15,18,19]. However, DOA doses should be restricted to a certain range because it reduces low- and medium-temperature performance. Like DOA, oil sands de-oiled asphalt (OSDOA), produced by the vacuum residue of oil sands asphalt, has a higher softening point and lower penetration. Its softening point exceeds 130 °C because of the massive enrichment of asphaltenes. The disposition of OSDOA has become a bottleneck for Canadian upgraders due to its enormous production and huge landfill costs. Applying OSDOA as a modifier is an effective way to reduce environmental pollution and cost.

Polyphosphoric acid (PPA) is an oligomer that is acquired by condensing mono-phosphoric acid or by hydrating phosphorous pentoxide (P_2_O_5_). It has become the most widely used acid in the technical field of modified asphalt because of its excellent modification effect, simple preparation technique, low price, and good compatibility with asphalt. The literature shows that a small amount of PPA can markedly enhance the Superpave performance grade (PG) of asphalt binders [20]. It takes approximately 0.8 wt% to 1.2 wt% PPA to raise a full high-temperature PG grade. Additionally, because PPA can enhance high-temperature rheological performances without degrading low-temperature properties, it can extend the range of high- and low-temperature properties of binders. The modification mechanism of PPA has not yet been conclusively determined because of the complexity and variability of the reactions in the modification process. However, one hypothesis is well recognized by researchers. It proposes that PPA reacts with the active functional group of asphalt, resulting in the breaking up of asphaltenes clusters and the dispersion of asphaltenes in maltene matrix [21]. The increase in the amount of asphaltenes mass and the enhancement of its dispersion effectively promote the viscoelastic behavior of asphalt [22].

Currently, PPA is commonly used with another polymer to modify asphalt to enhance its rheological properties. In this regard, the use of PPA and SBS to prepare composite modified asphalt is cost-effective. Zhang et al. [23,24] found that PPA enhanced the elevated temperature property of an SBS-modified binder, but declined its storage stability. The gelation of PPA resulted in severe phase separation between SBS and asphalt. Xiao et al. [25] showed that partial replacement of SBS with PPA is feasible to reduce viscosity. Applying 0.5 wt% PPA could reduce SBS by 1.0 wt% to prepare a PG 76–22 binder. Liu et al. [26] studied the elevated temperature rheological behavior and short-term anti-aging performance of SBS/PPA-modified binders. The results showed that the short-term anti-aging property of modified binder could be improved by PPA. Alam et al. [20] revealed the changes in chemical components of asphalt binders after adding PPA and SBS. They concluded that PPA gave rise to the asphaltenes contents and SBS changed the aromatics and resins contents. The combined use of PPA and SBS contributed to a sharp increase in asphaltenes concentration. Ramayya et al. [27] evaluated the effect of PPA on asphaltenes’ microstructure using X-ray diffraction and a scanning electron microscope. Experimental results showed that the incorporation of PPA effectively transformed the asphaltenes from a semi-crystalline structure to an amorphous structure. Consequently, PPA strengthens the elevated temperature properties of modified binder by increasing its asphaltenes content and changing its structure. This may provide inspiration for adding additional asphaltenes into neat asphalt and then modifying it with PPA to significantly improve the elastic performance. However, research regarding SBS/OSDOA/PPA-modified asphalt, which is vital to explore the modification mechanism for further application, is still rare and insufficient.

This research aims to solve the problem of OSDOA utilization in refineries and reduce its potential environmental pollution. The novelty of this study is to modify the SBS-modified binder with OSDOA and PPA to reduce the modification costs and improve the rheological properties and storage stability of composite modified asphalt. Therefore, the influences of OSDOA and PPA on the performance of SBS-modified binder were investigated, including its physical performance, rheological behavior, storage stability and microstructure. For this purpose, composite modified asphalts with a certain concentration of SBS and various contents of OSDOA and PPA were obtained. Meanwhile, the impacts of OSDOA and PPA on the properties of SBS-modified binder were also evaluated. Moreover, the optimal concentrations of OSDOA and PPA were recommended to prepare desirable composite modified asphalt with excellent high- and low-temperature properties and storage stability. Furthermore, the morphology and the variations in functional groups of asphalt were investigated.

## 2. Materials and Methods

### 2.1. Materials

**Figure 1:**
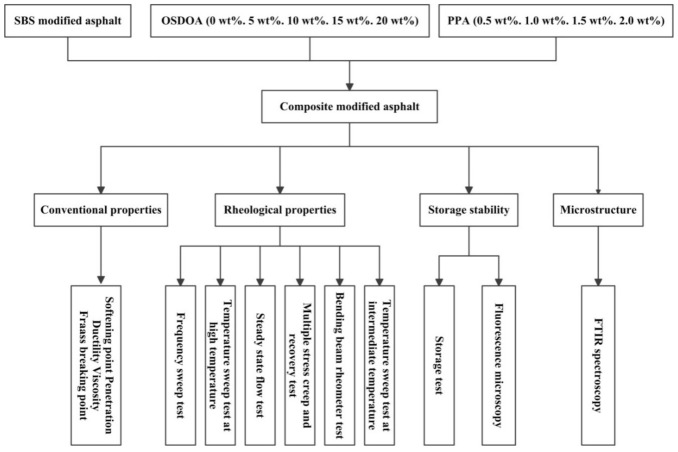
Technical roadmap of this research.

The neat asphalt applied in this study was from Karamay refinery in China with 80/100 penetration grade. Its main technical properties and chemical constituents are listed in Table 1. Liner SBS with 30 wt% styrene was purchased from Tianjin LG Chemical Co. Ltd., Tianjin, China; its basic properties are shown in Table 2. The OSDOA was prepared in the laboratory; its typical characteristics are presented in Table 3. PPA with a phosphorous pentoxide (P_2_O_5_) concentration greater than 85% was provided by Shanghai Aladdin Biochemical Technology Co. Ltd., Shanghai, China. Its technical indexes are displayed in Table 4. The technical roadmap of this study is presented in Figure 1.

### 2.2. Specimen Preparation

Composite modified asphalts were produced under high-speed shearing and stirring at high temperatures. First, the neat asphalt was heated to 160 °C in a batch reactor and then mixed with SBS (4 wt% base on neat asphalt) under a shear rate of 4000 rpm at 185 °C for 30 min. Afterward, sulfur (0.3 wt% base on neat asphalt) was slowly added to the blend and sheared for 20 min. Next, the OSDOA was then poured into the SBS-asphalt compound and stirred at 2000 rpm for 20 min at 185 °C. Five OSDOA concentrations (0 wt%, 5 wt%, 10 wt%, 15 wt%, 20 wt%) relative to the weight of the SBS-asphalt compound were investigated. For maximizing the use of OSDOA and ensuring the low-temperature property of the modified binder, the content of OSDOA was optimized to be 10 wt%. Finally, composite modified binders were produced by mixing the SBS/OSDOA-asphalt blend with a certain concentration of PPA. The proportions of PPA were 0.5 wt%, 1.0 wt%, 1.5 wt% and 2.0 wt% of SBS/OSDOA-asphalt. Then, the samples were gathered for the evaluation that followed. For the sake of simplicity, the composition and corresponding abbreviations of all modified asphalts are shown in Table 5.

### 2.3. Test Methods

#### 2.3.1. Conventional Performance Tests

The conventional performance tests, including penetration (25 °C), softening point and ductility (10 °C), were conducted in accordance with the technical specifications of modified asphalt specified in China’s Standard Test Methods of Bitumen and Bituminous Mixtures for Highway Engineering (JTG E20-2011) [33].

The viscosity of asphalt binder was measured by Brookfield rotational viscometer at 135 °C to investigate its construction workability. Furthermore, the Fraass breaking point was tested according to the experimental method in JTG E20-2011 to characterize its low-temperature performance.

#### 2.3.2. Rheological Property Tests

(1) DSR test

The dynamic shear rheometer (DSR) with parallel plate geometry was applied to test the rheological properties of the asphalt binders. A strain sweep test was performed on each specimen in advance to identify its linear viscoelastic range. To ensure the accuracy of test data, tests in different modes were repeated twice.

The frequency sweep test was carried on from 0.1 rad/s to 100 rad/s at 60 °C to obtain dynamic shear moduli. The temperature sweep test was performed at 10 rad/s as the temperature increases from 30 °C to 90 °C with 5 °C intervals. A viscous flow measurement with a setting test temperature of 60 °C was performed in the shear rate region between 10^−3^ s^−1^ and 10^2^ s^−1^.

(2) MSCR test

To detect the non-recoverable creep compliance and percent recovery of the asphalt binders, a multiple stress creep and recovery (MSCR) test was evaluated at 0.1 kPa and 3.2 kPa. The samples were aged in a rolling thin film oven (RTFO) and tested at 60 °C.

(3) BBR test

To determine the low-temperature anti-crack behavior of the modified asphalts, a bending beam rheometer (BBR) test was employed to measure the creep stiffness (S) and creep rate (m-value). The rolling thin film oven test (RTFOT)-pressure aging vessel (PAV) aged samples were characterized at −6, −12 and −18 °C.

#### 2.3.3. Storage Test

A high temperature storage test was performed to investigate the effects of OSDOA and PPA on the storage stability of the modified binder. An aluminum tube with a 2.5 cm diameter and 14 cm height was used to load approximately 50 g samples. Then, the tube was sealed and kept vertically in an oven for 48 h at 163 °C. After storage, the tube was cooled by refrigeration and divided into three equal parts. The dynamic shear moduli and softening points of the top and bottom portions were tested.

#### 2.3.4. Microscopic Morphology Tests

(1) FM test

Fluorescence Microscope (FM) is an effective method to observe the morphology of polymer modified asphalt. The dispersion of OSDOA and SBS in the asphalt was observed by fluorescence microscope Olympus BX51 (Tokyo, Japan). The magnification of the observation images was 400×.

(2) FTIR test

Fourier-transform infrared (FTIR) spectroscopy (Waltham, MA, USA) was used to characterize the variation in functional groups of modified asphalt. Furthermore, then the modification mechanism of composite modified asphalt was speculated. The wave number ranged from 650 cm^−1^ to 4000 cm^−1^.

## 3. Results

### 3.1. Conventional Properties

Table 6 lists the conventional performance indicators of composite modified asphalts with various contents of OSDOA and PPA. The table shows that 4 wt% SBS strengthens the softening point and reduces the penetration of neat asphalt. After OSDOA is added, the softening point and Brookfield viscosity increases, while penetration and ductility decreases. Additionally, the Fraass breaking point increases with an increasing concentration of OSDOA. The data show that OSDOA could upgrade the high-temperature performance but degrade the low-temperature property of asphalt binders because of its high stiffness [34,35]. To enable the maximum use of OSDOA and ensure the low-temperature performance of modified binder, the ductility ought to be more than 20 cm. Therefore, the optimized content of OSDOA is 10 wt%.

After PPA is incorporated, the softening point presents an increasing trend that rise dramatically when the PPA concentration exceeds 1.0 wt%. As can be seen, PPA also results in a marked increase in the Brookfield viscosity. Simultaneously, the penetration decreases with the increasing content of PPA because of its gelation character in asphalt. The results show that PPA provides an improving effect for the high-temperature properties of SBS/OSDOA-modified binder [27]. Moreover, low-temperature properties are also influenced by the amount of PPA. The ductility becomes even lower when PPA concentration exceeds 1.0 wt%. However, the Fraass breaking point remains basically unchanged, demonstrating that the low-temperature property of asphalt binder is little affected by PPA.

In conclusion, both OSDOA and PPA could strengthen the high-temperature performances of asphalt binders, while OSDOA reduces the low-temperature performances. The combination of OSDOA and PPA can effectively maximize the high-temperature property without impairing its low-temperature property. In the light of the Superpave specification, the viscosity of asphalt binder at 135 °C should be less than 3.0 Pa·s to ensure construction workability [36]. Hence, the optimal PPA content is 1.0 wt%.

### 3.2. Rheological Properties

#### 3.2.1. Frequency Sweep Tests

Asphalt binder is a viscoelastic material whose properties vary with loading time and temperature. The viscoelasticity of asphalt binder at low frequencies is analogous to the characteristics of pavement affected by slow traffic or high temperatures, whereas that at high frequencies is analogous to pavement affected by fast traffic or low temperatures. Thus, frequency sweep tests were conducted to characterize the effects of OSDOA and PPA on the rheology of SBS-modified asphalts.

**Figure 2:**
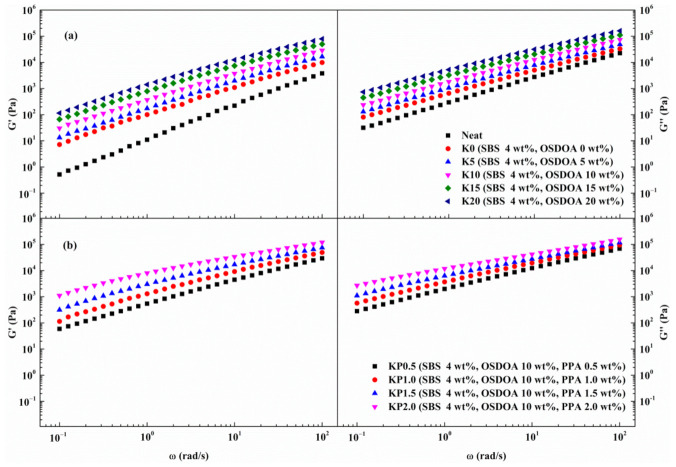
Variations in G′ and G″ with frequency: (**a**) SBS/OSDOA-modified asphalts; (**b**) composite modified asphalts.

Figure 2 presents the variations in shear storage modulus G′ and shear loss modulus G″ with frequency at 60 °C. It can be observed that both G′ and G″ enhance remarkably with increasing frequency, and the increment can reach several orders of magnitude. Apparently, the value of G″ is greater than that of G′ within the test frequency range, showing that viscous behavior plays the main role in the rheological properties. Moreover, the increase in frequency causes the gap between G′ and G″ to narrow, indicating that G′ is more sensitive to frequency. The addition of SBS significantly strengthens G′ and G″ compared with those of neat asphalt [37].

As observed from Figure 2, the G′ and G″ of composite modified binders are associated with the contents of OSDOA and PPA. Both G′ and G″ increase dramatically with the increased content of OSDOA. This result shows that the viscoelastic behavior of SBS-modified binder can be improved by OSDOA, which is attributable to the abundant asphaltenes in OSDOA. Moreover, the viscoelastic properties of SBS/OSDOA-modified binder are enhanced by a large margin after PPA is added. The increment of G′ is much greater than that of G″ as the PPA content increases. This suggests that the value of G′ is more sensitive to the concentration of PPA. The above results show that PPA could improve the concentration of the elastic component, which is conducive to improving the elastic properties of modified asphalts. This is because PPA could neutralize the polar interactions between stacked asphaltenes molecules, either by protonation or esterification. Consequently, PPA promotes the solvation of asphaltenes and raises the viscosity, thus enhancing the viscoelastic behavior of asphalt binder at high temperatures [20].

#### 3.2.2. Temperature Sweep Tests

**Figure 3:**
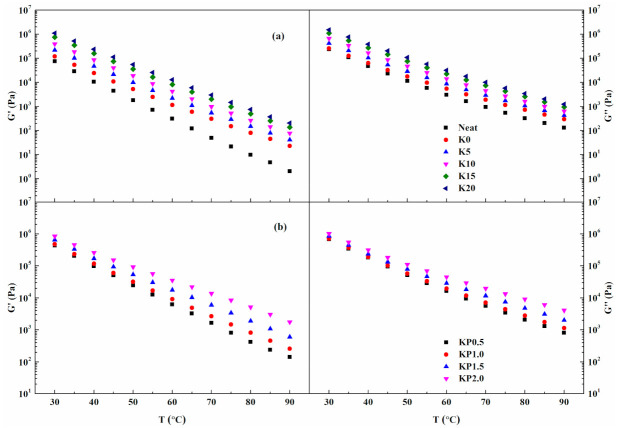
Variations in G′ and G″ with temperature: (**a**) SBS/OSDOA-modified asphalts; (**b**) composite modified asphalts.

The viscoelastic behavior of asphalt binder depends on temperature. Temperature sweep tests were performed to explore the impact of OSDOA and PPA on the viscoelastic properties of SBS-modified binder. The dynamic rheological properties of all binders under various temperatures are shown in Figure 3. As can be seen, G′ and G″ exhibit a decreasing trend with increasing temperatures. Moreover, the value of G″ is always higher than that of G′, showing that viscous behavior is dominant in the rheological properties. What is more, the decline range of G′ is greater than that of G″ as the temperature increases, especially at high temperatures. It suggests that higher temperatures contribute to the viscous behavior of the asphalt, corresponding with its classic rheological characteristics. Compared with base asphalt, G′ and G″ show a substantial increase after adding SBS, implying an enhanced viscoelastic performance of the asphalt.

The addition of OSDOA enhances both the G′ and G″, and the increment of G′ is greater than that of G″ with the increasing OSDOA content. The result suggests that OSDOA is beneficial to the enhancement of rheological properties, especially for the elastic behavior [38]. Moreover, the viscoelastic properties of OSDOA/SBS-modified asphalts are further strengthened by PPA. There is a prominent growth of G′ as PPA concentration raises, indicating that PPA could promote the elastic component of asphalt. Therefore, the combined effects of OSDOA and PPA substantially promote the rheological performances of the composite modified asphalts, particularly the elastic properties. It can be speculated that OSDOA and PPA could reinforce the anti-rutting performance of asphalt pavement.

**Figure 4:**
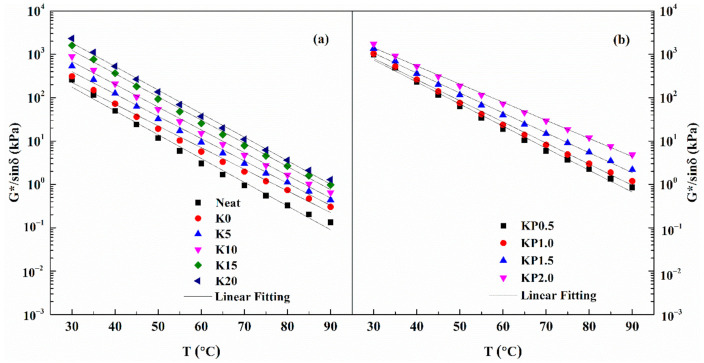
The fitting plots of G*/sinδ versus temperature for all samples: (**a**) SBS/OSDOA-modified asphalts; (**b**) composite modified asphalts.

The rutting resistance indicator G*/sinδ was developed to quantitatively describe the high-temperature performances of modified binder. Figure 4 shows the G*/sinδ values of all samples at various temperatures. Apparently, the rutting resistance index declines dramatically with the temperature increases. What is more, the values of G*/sinδ show an increasing trend as the OSDOA and PPA concentration increases. The failure temperature is determined to be when the G*/sinδ value is 1.0 kPa [39]. Failure temperatures of all samples are shown in Figure 4 and Table 7. As observed, the neat asphalt has the lowest failure temperature, and its failure temperature increases by 6 °C after SBS is added. The failure temperature steadily increases with increasing concentration of OSDOA, and it dramatically increases as the PPA content rise above 1.0 wt%. In other words, it is feasible to partially replace SBS with OSDOA and PPA. The concentration of OSDOA and PPA should be optimized through a comprehensive investigation of the composite modified binder’s properties at high and low temperatures.

#### 3.2.3. Viscous Flow Behavior

**Figure 5:**
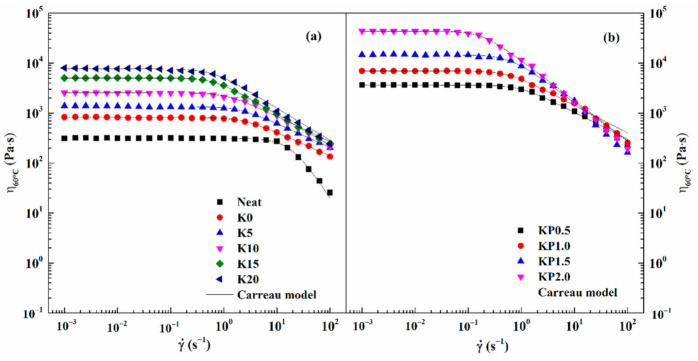
Carreau model fitting curves for all samples at 60 °C: (**a**) SBS/OSDOA-modified asphalts; (**b**) composite modified asphalts.

The steady flow viscosity with a shear rate approaching zero is referred to as zero shear viscosity (ZSV), which is an intrinsic property of asphalt binder [40]. It is based on the fact that asphalt binder tends to exhibit Newtonian fluid characteristics at high temperatures and extremely low shear rate, whereas the viscosities of Newtonian fluid are not affected by shear rate [41]. The ZSV can better characterize the rutting behavior of asphalt binder because the entire dissipative energy is a reflection of the viscous component or non-recoverable deformation [42,43]. Thus, the viscous flow behavior of all samples is evaluated at 60 °C; the flow curves are shown in Figure 5.

As shown in Figure 5, neat asphalt exhibits Newtonian fluid behavior over a wide shear rate range, and its viscosity remains constant within the shear rate region. In contrast, the viscosity of SBS-modified asphalt remains constant in the low shear rate region, and then the viscosity declines with the increasing shear rate, corresponding to a shear-thinning behavior. However, the shear-thinning phenomenon becomes more distinct as the OSDOA content increases. The presence of OSDOA and SBS leads to the transformation of the asphalt from classic Newtonian behavior to non-Newtonian behavior.

Moreover, a greater deviation from Newtonian behavior is observed after PPA is added, and the deviation is exacerbated by increased concentrations of PPA. The reason for the shear thinning may be that the SBS network structure is disrupted and orientated by shear stress, and the colloidal structure is transformed from “sol” type to “gel” type by adding OSDOA and PPA [11,12,21].

The viscous flow curves of all samples can be adequately described by Carreau model as follows:

(1) $$\eta =\frac{\eta_{0}}{{\left[1+{\left(\frac{\dot{\gamma }}{{\dot{\gamma }}_{c}}\right)}^{2}\right]}^{s}}$$

where $\eta_{0}$ represents zero shear rate viscosity, $\dot{\gamma_{c}}$ signifies the critical shear rate at the turning point of non-Newtonian behavior, and s is a parameter associated with the slope of the shear-thinning region.

Table 8 lists the fitting data of the Carreau model parameters. The table shows that the zero shear viscosity of neat asphalt is the lowest, and the viscosity greatly increases after SBS is added. The viscosity increases by several times with the addition of OSDOA, revealing that OSDOA contributes to the improvement in shear viscosity. The increase in OSDOA content leads to the improvement in viscosity. Moreover, the viscosity increases by an order of magnitude when the PPA concentration exceeds 1.0 wt%. The result demonstrates that PPA has the potential to significantly improve the resistance to the flowability of modified binder and to help reduce the occurrence of rutting distress on asphalt pavement. What is more, the critical shear rate transfers to a lower shear rate region and is more sensitive to shear stress, indicating that composite modified asphalt has more complicated and stronger interactions and entanglements among SBS, OSDOA, and PPA [44,45]. Therefore, OSDOA and PPA have a positive effect on the enhancement of the anti-flow behavior of asphalt pavement.

#### 3.2.4. Creep and Recovery Behavior

The Superpave specification parameter, G*/sinδ, seems inadequate to categorize modified binders [46,47]. MSCR test has been developed to evaluate high-temperature performances of polymer-modified binders by assessing the strain response under repeated shear loading and unloading at various stress levels [44,48,49]. Literatures show that the non-recoverable creep compliance of asphalt binder could better characterize the rutting potential of asphalt mixtures [50,51,52]. In order to better characterize the mechanical behavior of asphalt pavement, prediction constitutive models for rutting performance of flexible pavements were also proposed based on the viscoelastic properties of asphalt binder [53,54,55]. Thus, MSCR test was conducted to attain a thorough understanding of the creep and recovery behavior of composite modified asphalts.

**Figure 6:**
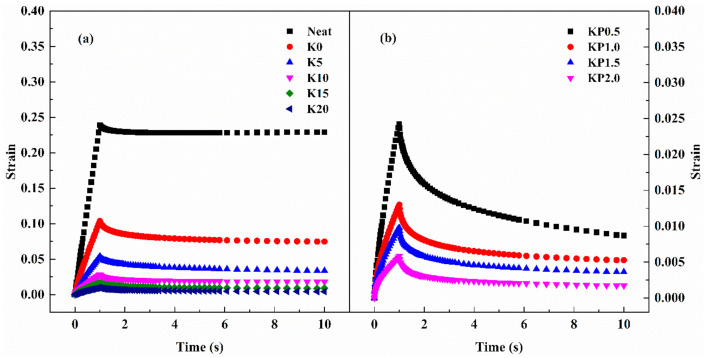
Typical one cycle of creep-recovery at 0.1 kPa and 60 °C: (**a**) SBS/OSDOA-modified asphalts; (**b**) composite modified asphalts.

RTFOT-aged samples were investigated by MSCR test at 60 °C; the strain responses at 0.1 kPa are shown in Figure 6. As can be observed, 1 s creep and 9 s recovery form a single cycle. In the creep process, the strain level increases with the increased loading time. During the recovery portion, as the loading stress is eliminated, the strain recovers immediately. Subsequently, it gradually declines to a constant strain value. Thus, a peak strain exhibits in the curve. These results reflect the viscoelastic behavior of asphalt. When the creep stress is unloaded, the strain of the elastic component recovers instantly, while that of the viscous component recovers gradually. The residual strain after recovery represents permanent deformation. As expected, the neat asphalt has the maximum residual strain value, while the composite modified binder containing 2.0 wt% PPA presents the minimum residual strain value. Above results reveal that the residual strain value is reduced by the addition of PPA.

**Figure 7:**
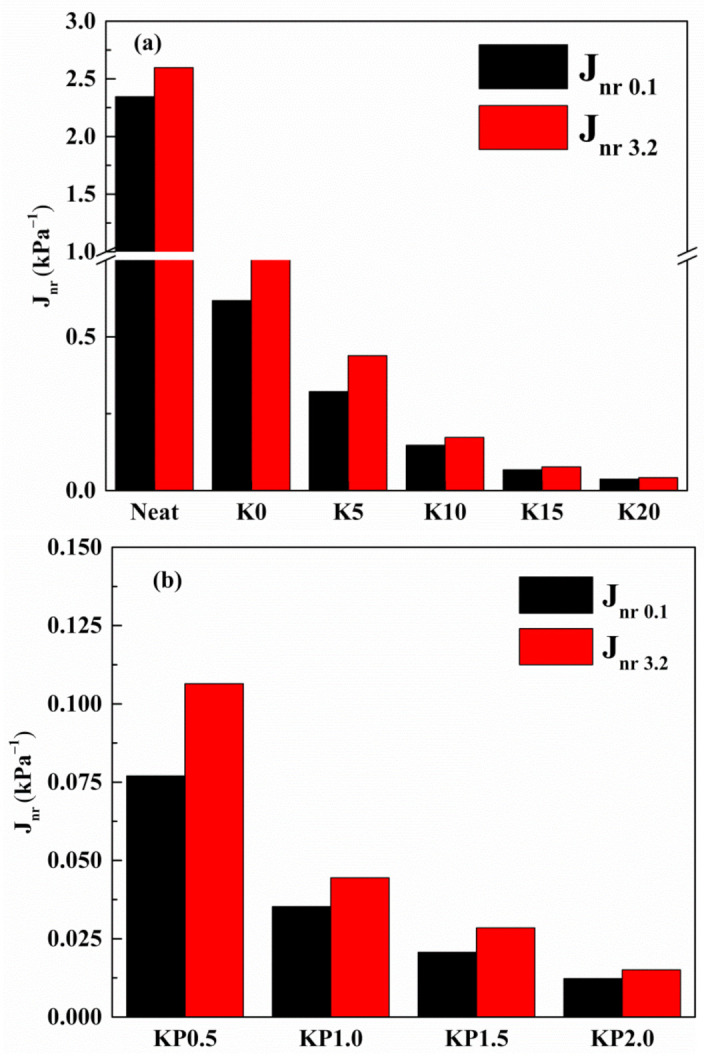
Average Jnr of tested samples under 0.1 kPa and 3.2 kPa at 60 °C: (**a**) SBS/OSDOA-modified asphalts; (**b**) composite modified asphalts.

**Figure 8:**
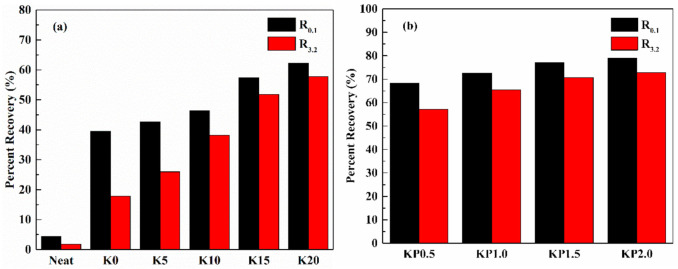
Average percent recovery of tested samples under 0.1 kPa and 3.2 kPa at 60 °C: (**a**) SBS/OSDOA-modified asphalts; (**b**) composite modified asphalts.

The effects of OSDOA and PPA on the high-temperature viscoelastic behavior of modified binders were quantitatively investigated, and the values of two vital parameters including average non-recoverable creep compliance (J_nr_) and average percent recovery (R) were obtained. The former represents the retained deformation of asphalt, and the latter characterizes the elastic properties of asphalt. A higher R value combined with a lower J_nr_ value indicates a superior anti-rutting performance of asphalt binder. Figure 7 and Figure 8 show the specific values of two parameters at 0.1 kPa and 3.2 kPa. Apparently, the addition of SBS enhances the R and declines the J_nr_ compared with those of the neat asphalt. After adding the OSDOA, the modified asphalt has a higher R and a lower J_nr_. It is noteworthy that the R value shows a gradually increasing trend with the increase in OSDOA content, while the J_nr_ value shows the opposite trend. These results suggest that OSDOA can enhance the elasticity of asphalt binder, thus promoting the deformation resistance of asphalt pavement. Moreover, the addition of PPA further increases the R value and decreases the J_nr_ value on the basis of OSDOA modification. What is more, J_nr_ increases, and R decreases with the increasing concentration of PPA. These results manifest that the elastic component of modified asphalt is enhanced by PPA, whereas the viscous component is diminished, which benefits the rutting resistance of asphalt pavement.

Furthermore, the applied stress level also has a major effect on the variation in R and J_nr_. The J_nr_ rises and the R drops dramatically with the increase in stress level, revealing that a higher stress level reduces the deformation resistance and the recovery properties of asphalt binder. Thus, rutting damage is prone to occur under heavy-duty traffic. Additionally, the variation in OSDOA and PPA concentrations contributes to the changes in R and J_nr_. For instance, for the variation in R, the R values of K0, K5, K10, K15, and K20 decrease by 55.0, 39.1, 17.7, 9.8, and 7.2%, respectively, due to the increase in stress level. In the same way, the R values of KP0.5, KP1.0, KP1.5, and KP2.0 drop by 16.2, 9.7, 8.4, and 7.8%, respectively. On the basis of the above results, it can be speculated that OSDOA and PPA can effectively mitigate the negative effect of the increased stress level [44].

#### 3.2.5. Low-Temperature Creep Behavior

**Figure 9:**
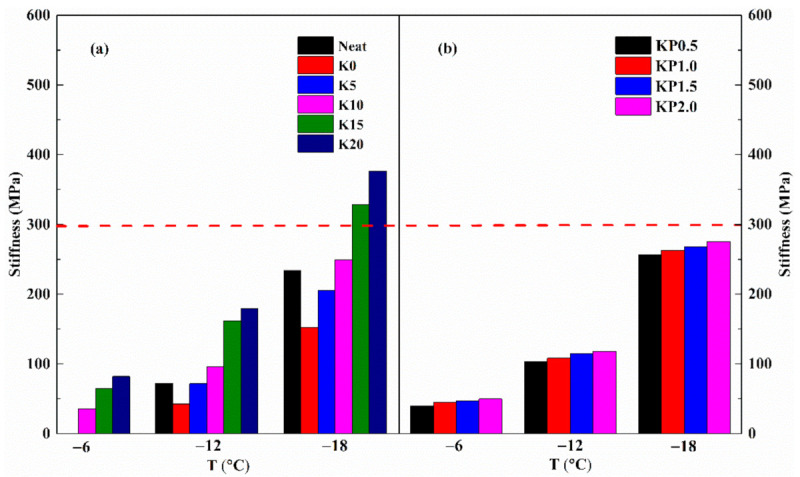
Variations in creep stiffness with temperature: (**a**) SBS/OSDOA-modified asphalts; (**b**) composite modified asphalts.

**Figure 10:**
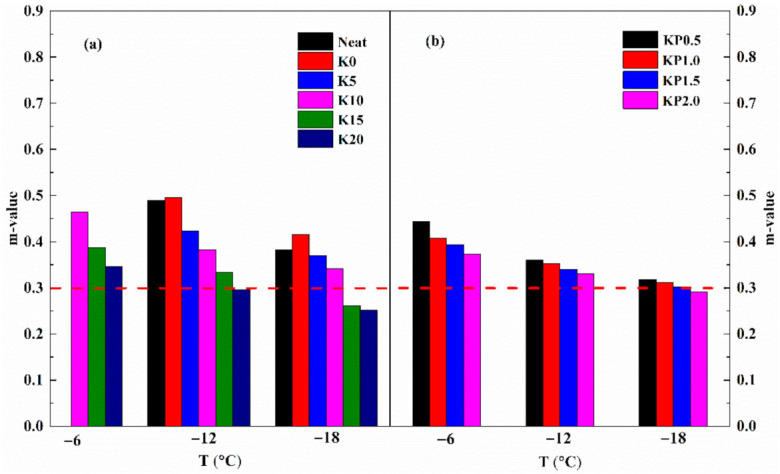
Variations in m-value with temperature: (**a**) SBS/OSDOA-modified asphalts; (**b**) composite modified asphalts.

As previously mentioned, the compound modification of OSDOA and PPA is helpful to enhance the high-temperature rheological behavior of modified binder. It is acknowledged that a widened range of high- and low-temperature performances is beneficial to extend pavement service life. In view of this, variations in low-temperature creep behavior caused by OSDOA and PPA were evaluated by BBR test at −6, −12 and −18 °C. Figure 9 and Figure 10 display the creep stiffness (S) and creep rate (m-value) of all tested samples.

It is generally believed that a lower stiffness and a higher m-value contribute to the prominent flexibility of asphalt at low temperatures. As observed, after SBS is added, the creep stiffness of neat asphalt decreases and the m-value increases, figuring that SBS makes the asphalt more flexible at low temperatures. The neat asphalt, K0 and K5 are too flexible to be detected at −6 °C. It is clear that as the OSDOA content increases, the stiffness value rises sharply and the m-value declines rapidly [38]. The results reveal that OSDOA has a detrimental effect on the low-temperature properties. Similarly, PPA also strengthens the stiffness and lowers the m-value, revealing that PPA hardens the binder and reduces its low-temperature anti-crack performance. Fortunately, the low-temperature performance of modified binder is only slightly affected by PPA, and the low-temperature grade remains unchanged even when the PPA concentration reaches 2.0 wt%. Therefore, an appropriate amount of PPA can guarantee the low-temperature grade of asphalt binder. Furthermore, temperature is a vital factor affecting low-temperature creep behavior. Distinct variations in creep stiffness and m-value appear as the temperature declines, showing the higher sensitivity of the modified binder to temperature. Hence, OSDOA and PPA proportions should be restricted in a certain range to optimize the low-temperature property of composite modified binder.

To meet the low-temperature anti-crack requirement, Superpave specifications require a stiffness lower than 300 MPa and an m-value higher than 0.3 [56]. Clearly, SBS/OSDOA-modified asphalts cannot reach the specification requirements at −18 °C when the OSDOA concentration exceeds 10 wt%. Thus, 10 wt% concentration of OSDOA was selected to ensure the low-temperature performance. What is more, the PPA hardly affects the low-temperature grading of the modified binder when the dose of PPA is less than 1.5 wt%. Within that range, all composite modified asphalts fulfill the minimum requirement at −18 °C. The low-temperature PG grade of asphalt is 10 °C lower than the BBR test temperature, and the low-temperature PG grade of the composite modified binder can reach −28 °C. Therefore, the best content of OSDOA is 10 wt%, and the proportion of PPA should be less than 1.5 wt% to achieve the low-temperature grade.

#### 3.2.6. Fatigue Behavior

**Figure 11:**
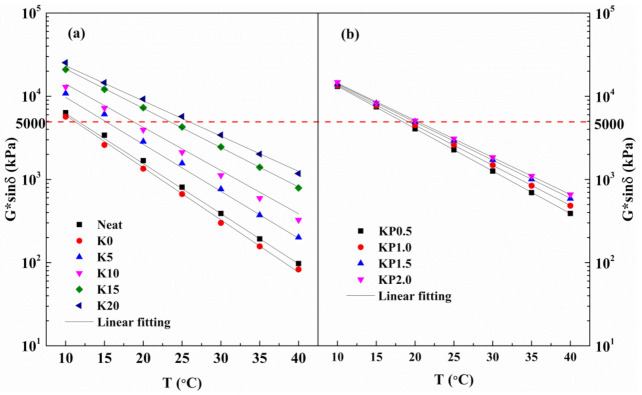
Temperature dependence of G*sinδ for all tested samples: (**a**) SBS/OSDOA modified asphalts; (**b**) composite modified asphalts.

Fatigue cracking, the most common failure of asphalt pavement, is caused by repeated traffic loading and asphalt aging. Repeated vehicle loading generates tensile stress at the pavement underlayer, and aging reduces the adhesion of asphalt binder, which leads to the formation of microcracks [57,58]. The further expansion of microcracks leads to the formation of fatigue cracks [59]. The literature shows that the fatigue performances of asphalt mixtures depend mainly upon the fatigue resistance of asphalt binders [60]. The Strategic Highway Research Program (SHRP) employs G*sinδ as the fatigue index to assess a binder’s fatigue resistance based on its linear viscoelastic performance. Therefore, the fatigue behavior of all tested samples was evaluated by temperature sweep test; the values of G*sinδ are presented in Figure 11.

As shown in the figure, the values of G*sinδ are reduced as the temperature increases. It is generally believed that a lower G*sinδ indicates better fatigue resistance. As can be seen, the values of G*sinδ rise with the increasing OSDOA content, indicating that OSDOA is harmful to the fatigue property of the asphalt binder. Similarly, PPA also increases the values of G*sinδ. Therefore, the combination of OSDOA and PPA reduces the fatigue resistance of the asphalt binder.

The fatigue parameter G*sinδ should be less than 5000 kPa to prevent fatigue cracking [61]. The fatigue temperatures are calculated when the G*sinδ is equal to 5000 kPa. Table 9 displays the specific values. It is found that the fatigue temperatures increase as the OSDOA content increases, implying that OSDOA can reduce the fatigue resistance performance of asphalts. The fatigue temperatures increase remarkably as the OSDOA content exceeds 10 wt%, revealing that OSDOA should be limited to a certain concentration range. Likewise, fatigue temperatures also increase with the increase in PPA content, although the increment is comparatively small. The results suggest that the effect of OSDOA on the anti-fatigue performance of asphalt is far greater than that of PPA. In conclusion, the anti-fatigue properties of composite modified binders are degraded by adding OSDOA and PPA, and the decrement depends on both OSDOA and PPA concentrations.

### 3.3. Storage Stability

Storage stability is a quite critical technical criterion in the production, transportation, and storage of modified asphalt [62]. However, phase separation may occur during storage at high temperatures due to discrepancies in the density, molecular structure, and solubility parameter between polymers and neat asphalt. The phase separation results in degradation of the rutting performance, low-temperature flexibility, and fatigue property of modified asphalt, which limits the practical application of modified asphalt [63,64]. Thus, the effects of OSDOA and PPA on the storage stability of composite modified binders were studied by softening-point differences and viscoelastic performance differences.

Variation in the softening point can directly reflect the massive phase separation phenomenon of modified binder. More importantly, the detection method of softening point is simple and reliable. Table 10 presents the values of softening-point differences between the top and bottom portions of modified binders. To guarantee the high-temperature storage stability of modified binder, it is generally believed that the softening-point difference should be less than 2.5 °C. The softening-point difference of K0 reaches 4.5 °C, indicating that SBS floats to the top of the tube and asphaltenes precipitate to the bottom. After 5 wt% of OSDOA is added, the difference becomes less than 2.5 °C, revealing that K5 has superior storage stability. However, the difference in softening point becomes greater with an increased OSDOA content, which is associated with serious phase separation taking place in the modified asphalt. These results demonstrate that the appropriate amount of OSDOA could improve storage stability, while excessive OSDOA could cause binder separation. This is because greater asphaltenes content tends to cause a phase separation of the modified binder [63]. However, the addition of PPA reduces the softening-point difference, revealing that PPA could mitigate the severe phase separation caused by the OSDOA and improve high-temperature storage stability. What is more, the softening-point difference of the composite modified binder is less than 2.5 °C when the PPA concentration exceeds 0.5 wt%. Such a phenomenon demonstrates that PPA could strengthen the compatibility of SBS/OSDOA-modified binder. Consequently, PPA content should be greater than 0.5 wt% to ensure storage stability.

The rheological method is sensitive to the microstructure and viscoelastic changes in a modified binder. Separation index I_s_ was developed by the SHRP to identify the separation degree; it was defined as follows [11]:

(2) $$I_{s}=\log(G_{b}^{*}/G_{t}^{*})$$

where $G_{b}^{*}$ and $G_{t}^{*}$ correspond to the dynamic shear modulus of the bottom and top parts at 60 °C and 10 rad/s, respectively.

**Figure 12:**
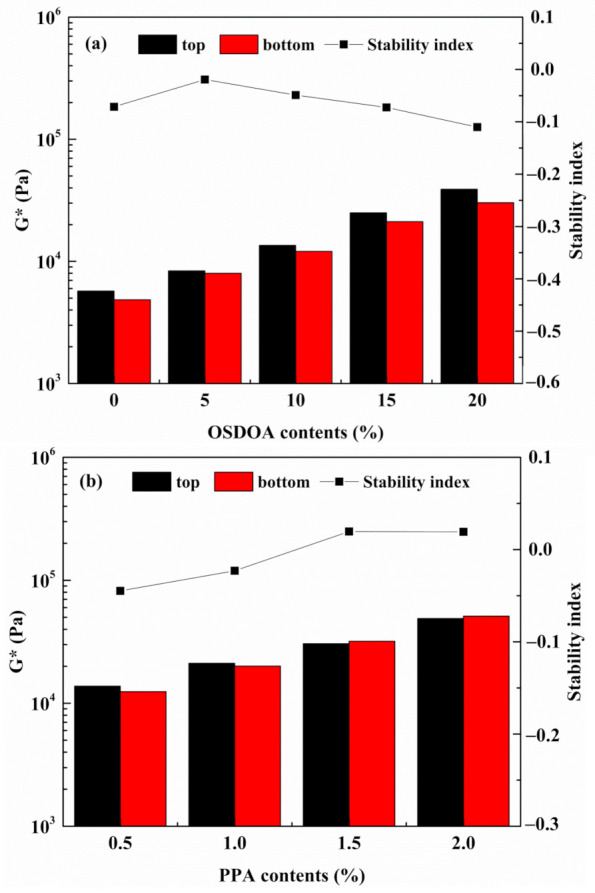
Variations in G* and the stability index with various OSDOA and PPA contents: (**a**) SBS/OSDOA-modified asphalts; (**b**) composite modified asphalts.

An I_s_ value close to zero indicates superior storage stability. The values of the dynamic shear moduli of the two fractions after high-temperature storage are displayed in Figure 12. As observed, the gap between $G_{b}^{*}$ and $G_{t}^{*}$ becomes narrow first and then becomes wider as the content of OSDOA increases. Similarly, the value of I_s_ approaches zero first and then moves away from zero. After PPA is added, the difference between $G_{b}^{*}$ and $G_{t}^{*}$ declines with the increase in PPA concentration. Meanwhile, the I_s_ values tend to zero by adding PPA, indicating that PPA helps enhance the storage stability. Thus, the viscoelastic performance is consistent with the results of softening-point differences, confirming that PPA benefits storage stability [45].

**Figure 13:**
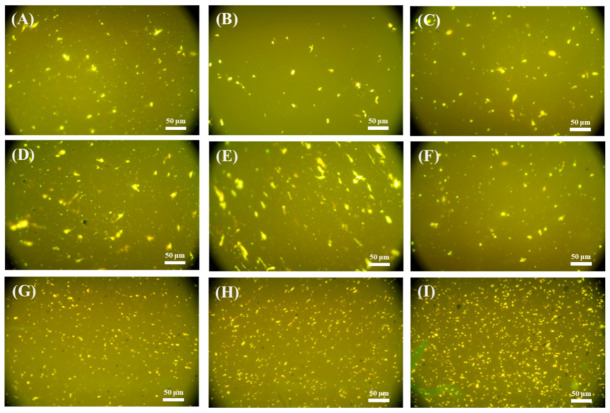
Fluorescence microscopy of all samples (×400): (**A**) K0; (**B**) K5; (**C**) K10; (**D**) K15; (**E**) K20; (**F**) KP0.5; (**G**) KP1.0; (**H**) KP1.5; (**I**) KP2.0.

The morphology of the composite modified asphalt is characterized to observe the microstructural changes. Fluorescence images of SBS-modified asphalt, SBS/OSDOA-modified asphalts, and composite modified asphalts are displayed in Figure 13. In these images, the bright yellow phase represents swollen SBS particles, and the dark region indicates the asphalt phase. Figure 13A reveals that the swollen SBS polymers are scattered in the asphalt phase with irregular shapes, indicating the incompatibility between SBS and asphalt. Such morphology suggests that phase separation may occur. As depicted in Figure 13B, the SBS particles become smaller and more dispersed in the asphalt phase after 5 wt% OSDOA is added. However, the SBS particles become larger and aggregated with the increase in OSDOA concentration, as displayed in Figure 13C–E. This is because OSDOA and SBS compete for the absorption of maltenes, leading to the precipitation and coalescence of SBS droplets from the asphalt phase. Additionally, marked variations in the SBS dispersed state can be observed after PPA is added, the particles become much smaller, and the dispersion is more uniform, as shown in Figure 13F–I. It implies that PPA could keep the modified asphalt more stable stored at a high temperature. Hanyu et al. [65] showed that greater fineness and better dispersion of the polymer can improve storage stability. Liang et al. [45] also revealed that PPA could reduce the particle size of the polymer in asphalt matrix and promote its more uniform dispersion. Consequently, PPA could promote the dispersion of SBS particles in asphalt, thus enhancing the storage stability of the composite modified binder.

### 3.4. Microstructure

**Figure 14:**
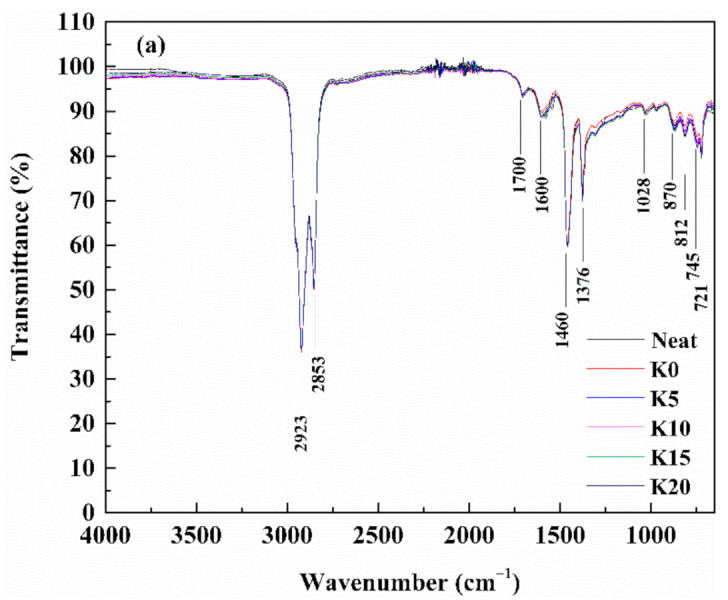

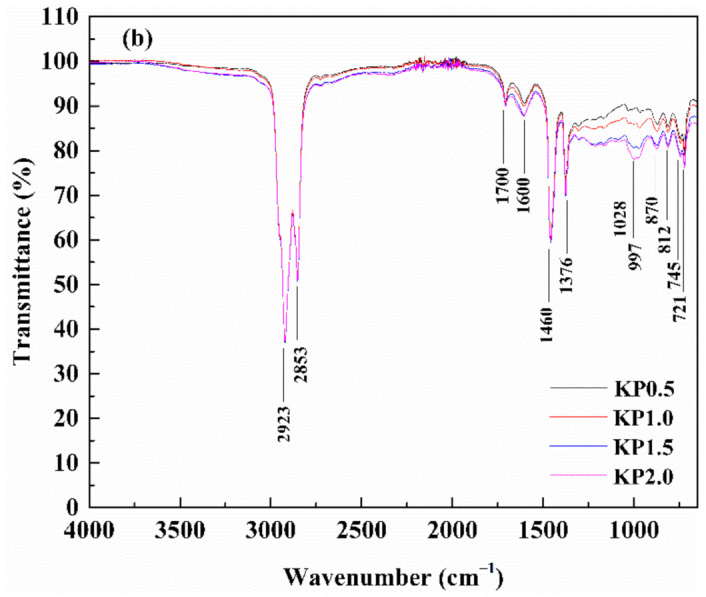
FTIR spectra of tested samples: (**a**) SBS/OSDOA-modified asphalts; (**b**) composite modified asphalts.

It can be known from the above results that the rheological properties and morphology of composite modified binder change after OSDOA and PPA are added. FTIR spectroscopy has been demonstrated to be an effective and convenient approach to analyze the chemical and structural variations in the process of modification by identifying different absorption peaks [8,66,67]. Therefore, FTIR characterization of composited modified asphalt was evaluated in order to explore the modification mechanism. Figure 14 displays the major absorption peaks of all binders.

As observed, the classic absorption bands for all samples are located mainly at 2923, 2853, 1700, 1600, 1460, 1376, 1028, 870, 812, 745, and 721 cm^−1^. The strong absorption peaks at 2923 and 2853 cm^−1^ are due to the C-H stretching vibrations of aliphatic hydrogen. The stretching vibrations of C=O and C=C appear at 1700 and 1600 cm^−1^, respectively. The absorption bands at 1460 and 1376 cm^−1^ are ascribed to the C-H bending vibrations. The stretching vibration of S=O shows at 1028 cm^−1^. The weak absorption bands distributed in the 700–900 cm^−1^ region are caused by the C-H bending vibrations of aromatic ring.

Figure 14 shows that no new peak appears in the FTIR spectra of SBS/OSDOA-modified asphalts, indicating that OSDOA modification is a mainly physical reaction. However, a new absorption peak appeared at 997 cm^−1^ for the composite modified binder, which corresponded to the stretching vibrations of P-O [68,69]. This indicates that interactions have occurred between PPA and asphalt that lead to the microstructural changes in the modified asphalt. Ge et al. [70] found that some functional groups were produced and some absorption peaks vanished after the addition of PPA. Masson et al. [71,72,73,74] revealed that some functional groups in the asphalt binder may react with PPA and produce phosphorylated products. These results demonstrate that PPA chemically interacts with the asphalt binder. As a consequence, physical and chemical interactions coexist during the OSDOA/PPA modification process.

## 4. Conclusions

To strengthen the high- and low-temperature properties and storage stability of SBS-modified binder at a lower cost, OSDOA and PPA were utilized to further modify the binder. The effects of OSDOA and PPA on physical properties, viscoelastic performance, storage stability, and microstructure of the SBS-modified binder were comprehensively evaluated. The primary conclusions can be summarized as follows:

(1) The compound modification of OSDOA/PPA on SBS-modified asphalt markedly improves the resistance to permanent deformation. Furthermore, the low-temperature PG grade of composite modified binder can be guaranteed to reach −28 °C by optimizing the concentration of OSDOA and PPA.
(2) Fatigue resistance of SBS-modified asphalt is degraded by the compound modification of OSDOA and PPA. Moreover, the effect of OSDOA on the fatigue behavior of SBS-modified binder is far greater than that of PPA.
(3) The composite modified asphalt has excellent storage stability, which is verified by fluorescence images. FTIR analysis reveals that physical and chemical interactions coexist during the OSDOA/PPA modification process.
(4) By a comprehensive consideration of the balance of high- and low-temperature properties and storage stability, the optimal doses of OSDOA and PPA are determined to be 10 wt% and 1.0 wt%, respectively.

## Figures and Tables

**Table 1:** Physical and chemical properties of neat asphalt.

Item	Neat Asphalt	Specifications
Softening point (°C)	47.2	ASTM D36 [28]
Penetration (25 °C, 0.1 mm)	87	ASTM D5 [29]
Ductility (10 °C, cm)	>100	ASTM D113 [30]
Viscosity (135 °C, Pa·s)	0.595	ASTM D4402 [31]
Saturates (wt%)	28.63	ASTM D4124 [32]
Aromatics (wt%)	34.04	ASTM D4124
Resins (wt%)	37.21	ASTM D4124
Asphaltenes (wt%)	0.12	ASTM D4124

**Table 2:** Basic properties of SBS.

Item	SBS
Density (g/cm^3^)	0.94
Melt Flow Rate (g/10 min)	<0.5
Styrene to Butadiene	30/70
Elongation at Break (%)	750
Tensile Strength (MPa)	24
Hardness	76
Structure	Linear

**Table 3:** Physical and chemical properties of OSDOA.

Item	OSDOA	Specifications
Softening point (°C)	135	ASTM D36
Penetration (25 °C, 0.1 mm)	0	ASTM D5
Saturates (wt%)	3.45	ASTM D4124
Aromatics (wt%)	23.03	ASTM D4124
Resins (wt%)	34.48	ASTM D4124
Asphaltenes (wt%)	39.04	ASTM D4124

**Table 4:** Technical indexes of PPA.

Item	PPA
Concentration of P_2_O_5_ (%)	>85
Density (g/cm^3^)	2.06
Molecular weight	337.9
Boiling point (°C)	300
Specific heat (J/g·°C)	1.487

**Table 5:** The composition and corresponding abbreviations of all modified binders.

Item	SBS (wt%)	OSDOA (wt%)	PPA (wt%)
K0	4	0	0
K5		5	0
K10		10	0
K15		15	0
K20		20	0
KP0.5		10	0.5
KP1.0		10	1.0
KP1.5		10	1.5
KP2.0		10	2.0

**Table 6:** Conventional properties of modified binders.

Item	Penetration (25 °C, 0.1 mm)	Softening point (°C)	Ductility (10 °C, cm)	Viscosity (135 °C, Pa·s)	Fraass Breaking Point (°C)
K0	73	55.4	60	0.127	−14
K5	50	58.4	35	0.167	−13
K10	38	63.6	22	2.005	−12
K15	28	67.2	10	2.730	−11
K20	24	69.0	5	3.365	−10
KP0.5	37	64.6	20	2.480	−12
KP1.0	35	67.8	14	2.915	−13
KP1.5	30	73.2	10	5.963	−13
KP2.0	26	80.5	8	9.538	−13

**Table 7:** Failure temperatures of all tested specimens.

Samples	Failure Temperature (°C)
Neat	70.95
K0	77.00
K5	80.57
K10	83.94
K15	87.79
K20	90.08
KP0.5	86.50
KP1.0	89.68
KP1.5	95.98
KP2.0	105.32

**Table 8:** Parameter values of Carreau model.

Samples	$\eta_{0}\;(\mathrm{Pa}\text{·}s)$	$\dot{\gamma_{c}}\;(s^{-1})$	s
Neat	303.89	25.24	0.4942
K0	1351.96	0.10	0.1219
K5	2037.21	0.22	0.1476
K10	3546.26	0.21	0.1616
K15	5255.00	0.18	0.1667
K20	8540.31	0.15	0.1799
KP0.5	5340.60	0.11	0.1645
KP1.0	7663.38	0.08	0.1682
KP1.5	16,429.90	0.07	0.2083
KP2.0	48,136.47	0.05	0.2518

**Table 9:** Fatigue temperatures of all tested specimens.

Samples	Fatigue Temperature (°C)
Neat	11.99
K0	10.64
K5	16.05
K10	17.91
K15	23.30
K20	26.02
KP0.5	18.29
KP1.0	19.04
KP1.5	19.79
KP2.0	20.27

**Table 10:** Softening point differences of modified asphalt binders.

Samples	Softening Point (°C)		Differences (°C)
	Top	Bottom	
K0	59.4	54.9	4.5
K5	59.0	58.4	0.6
K10	64.4	61.0	3.4
K15	71.4	66.7	4.7
K20	77.5	68.6	8.9
KP0.5	71.3	68.6	2.7
KP1.0	74.1	73.1	1.0
KP1.5	77.7	78.4	−0.7
KP2.0	82.9	83.5	−0.6

## Data Availability

The data presented in this study are available on request from the corresponding author.
